# Microtissue Culture Provides Clarity on the Relative Chondrogenic and Hypertrophic Response of Bone-Marrow-Derived Stromal Cells to TGF-β1, BMP-2, and GDF-5

**DOI:** 10.3390/cells13010037

**Published:** 2023-12-23

**Authors:** Rose Ann G. Franco, Eamonn McKenna, Md. Shafiullah Shajib, Bianca Guillesser, Pamela G. Robey, Ross W. Crawford, Michael R. Doran, Kathryn Futrega

**Affiliations:** 1 Centre for Biomedical Technologies (CBT), School of Mechanical, Medical and Process Engineering, Faculty of Engineering, Queensland University of Technology (QUT), Brisbane, QLD 4000, Australia; 2 Translational Research Institute (TRI), Brisbane, QLD 4102, Australia; 3 School of Biomedical Science, Faculty of Health, Queensland University of Technology (QUT), Brisbane, QLD 4000, Australia; 4 Skeletal Biology Section, National Institute of Dental and Craniofacial Research (NIDCR), National Institutes of Health (NIH), Department of Health and Human Services, Bethesda, MD 20892, USA; 5 Mater Research Institute—University of Queensland (UQ), Translational Research Institute (TRI), Brisbane, QLD 4102, Australia

**Keywords:** TGF-β1, BMP-2, GDF-5, BMSC chondrogenesis, hypertrophy, Microwell-mesh

## Abstract

Chondrogenic induction of bone-marrow-derived stromal cells (BMSCs) is typically accomplished with medium supplemented with growth factors (GF) from the transforming growth factor-beta (TGF-β)/bone morphogenetic factor (BMP) superfamily. In a previous study, we demonstrated that brief (1–3 days) stimulation with TGF-β1 was sufficient to drive chondrogenesis and hypertrophy using small-diameter microtissues generated from 5000 BMSC each. This biology is obfuscated in typical large-diameter pellet cultures, which suffer radial heterogeneity. Here, we investigated if brief stimulation (2 days) of BMSC microtissues with BMP-2 (100 ng/mL) or growth/differentiation factor (GDF-5, 100 ng/mL) was also sufficient to induce chondrogenic differentiation, in a manner comparable to TGF-β1 (10 ng/mL). Like TGF-β1, BMP-2 and GDF-5 are reported to stimulate chondrogenic differentiation of BMSCs, but the effects of transient or brief use in culture have not been explored. Hypertrophy is an unwanted outcome in BMSC chondrogenic differentiation that renders engineered tissues unsuitable for use in clinical cartilage repair. Using three BMSC donors, we observed that all GFs facilitated chondrogenesis, although the efficiency and the necessary duration of stimulation differed. Microtissues treated with 2 days or 14 days of TGF-β1 were both superior at producing extracellular matrix and expression of chondrogenic gene markers compared to BMP-2 and GDF-5 with the same exposure times. Hypertrophic markers increased proportionally with chondrogenic differentiation, suggesting that these processes are intertwined for all three GFs. The rapid action, or “temporal potency”, of these GFs to induce BMSC chondrogenesis was found to be as follows: TGF-β1 > BMP-2 > GDF-5. Whether briefly or continuously supplied in culture, TGF-β1 was the most potent GF for inducing chondrogenesis in BMSCs.

## 1. Introduction

Bone-marrow-derived stromal cells (BMSCs), which are easily isolated and can be expanded in vitro, are a cell population that could be exploited in cartilage repair. Most chondrogenic induction protocols mimic aspects of a 1990s method where BMSCs were aggregated into a pellet and cultured for two to three weeks in differentiation media supplemented with transforming growth factor-beta 1 (TGF-β1) [1]. Unfortunately, BMSCs have a propensity to undergo hypertrophic differentiation, and thus far, there is no widely reproduced differentiation protocol that enables the generation of stable chondrocyte-like cells suitable for use in cartilage defect repair [2,3].

During embryonic skeletal development, cell types evolve in response to growth factor signals delivered in a defined spatiotemporal manner [4], and there may be merit in attempting to mimic temporal dosing during BMSC chondrogenic induction. For such a process to work, it is presumed that cells differentiate in a step-wise fashion in vitro, where following condensation, cells first take on a chondrogenic phenotype but can enter a terminal stage of hypertrophy if improperly stimulated, thus developing undesirable bone-like features [5]. In contradiction to this presumed paradigm, we recently discovered that a single day of TGF-β1 exposure triggers both BMSC chondrogenic and hypertrophic differentiation, and that subsequent differentiation is driven by intrinsic signalling cascades that continue concurrently independent of further TGF-β1 stimulation [6]. Effectively, all instruction is laid down during the first day of TGF-β1 stimulation. After this first day, intrinsic signalling drives both chondrogenic and hypertrophic differentiation in a parallel and intertwined manner over the subsequent 14–21 days. This behaviour suggests that BMSC, at least in response to TGF-β1 stimulation, do not respond to temporal growth factor stimulation in an incremental stepwise manner from condensation to a chondrocyte-like cell to hypertrophic chondrocyte. We reason that BMSC response to TGF-β1 stimulation has been overlooked because the patterns are obfuscated within the heterogenous tissue that evolves in typical large-diameter pellet cultures. We performed parallel experiments with large-diameter pellet cultures and small-diameter microtissues, and these complex patterns were only clearly discernible in small-diameter microtissues. Knut et al. also reported that treating cultures with TGF-β1 for 7 days instead of the whole duration of a 28-day culture was sufficient to induce chondrogenesis in human MSC micropellets [7]; these data also suggest that brief TGF-β1 stimulation is sufficient to trigger differentiation programs, but that the phenomenon was only visible when initially triggered in small-diameter micropellets. Having gained a new understanding of how TGF-β1 stimulation influences BMSC differentiation, and an appreciation for the role of small-diameter microtissues in deconvoluting this understanding, we sought to characterise how stimulation with other common chondrogenic induction factors, bone morphogenetic factor-2 (BMP-2), and growth/differentiation factor-5 (GDF-5), influenced BMSC differentiation in a microtissue model.

TGF-β1, BMP-2, and GDF-5 are all logical factors to explore in efforts to generate cartilage-like tissue from BMSC. TGF-βs play a crucial role in mesenchymal condensation, proliferation, and the expression of genes, which influence glycosaminoglycan (GAG) production and terminal differentiation during development as well as cartilage maintenance postnatally [8]. Like TGF-βs, BMPs and GDFs are subsets of the TGF-β/BMP superfamily, and they also play an important role in mesenchymal condensation during early chondrogenesis, proliferation, and hypertrophy [9,10]. Supplementation of BMSC chondrogenic induction cultures with BMP-2, -4, or -6 was previously shown to significantly boost extracellular matrix production, with BMP-2 having the greatest effect [11]. BMP-2 has been shown to induce chondrogenesis by stimulating GAG production and chondrogenic gene expression in human adult BMSC [12], synovial bovine explants [13], human embryonic stem cells [14], and adipose-derived stromal cells [15]. During development in mice, GDF-5-expressing cells mark regions of joint specification and contribute to all synovial joint components, including articular cartilage [16]. GDF-5 has been previously used to induce chondrogenesis in cultures of rabbit BMSCs [17], rat adipose stromal cells [18], human BMSC pellet cultures [19], and human chondrocytes [20] and aided in the suppression of cell death in the presence of TGF-β1 in human embryonic stem-cell-derived chondrocytes [14].

Herein, we asked if BMSCs respond to BMP-2 or GDF-5 in a manner similar to TGF-β1, where brief stimulation initiates an intrinsic signalling cascade that directs subsequent differentiation. We used 10 ng/mL of TGF-β1 based on our previous studies [6,21,22,23,24] and 100 ng/mL of BMP-2 or GDF-5 following methodologies from previous literature [17,18,25,26,27,28,29,30,31]. BMSCs were assembled into microtissues and cultured in the Microwell-mesh, a high-throughput microtissue culture platform developed in our laboratory [23]. We evaluated the quality of chondrogenic induction based on relative tissue growth (microtissue diameter, DNA content, and relative metabolic activity), GAG production, chondrogenic gene expression, and histology.

## 2. Materials and Methods

### 2.1. BMSC Isolation, Expansion, Flow Cytometry Characterisation, and Chondrogenic Induction

BMSCs were isolated as described previously [23] using protocols approved by the Mater Hospital Human Research Ethics Committee and in accordance with the National Health and Medical Research Council of Australia guidelines (Ethics number: 1541A). In brief, BMSC cultures were established from 20 mL of heparinised bone marrow aspirate collected from the iliac crests of consenting healthy volunteer adult human donors at the Mater Hospital, Brisbane, Australia. Using Ficoll-Paque PLUS (GE Healthcare, Marlborough, MA, USA) density gradient centrifugation, mononuclear cells were enriched from the bone marrow aspirate. Mononuclear cells were distributed into five T175 culture flasks, each in 35 mL of expansion medium. Expansion medium was formulated from low glucose Dulbecco’s Modified Eagle Medium (LG-DMEM) supplemented with 10% fetal bovine serum (FBS, Thermo Fisher Scientific, Waltham, MA, USA), 1% penicillin/streptomycin (PenStrep, Gibco, Thermo Fisher Scientific), 10 ng/mL of fibroblast growth factor-1 (FGF-1, PeproTech, Cranbury, NJ, USA), and 5 µg/mL of heparin (Sigma-Aldrich, St. Louis, MO, USA). Flasks were placed into a 20% O_2_, 5% CO_2_, and 37 °C incubator overnight to allow the cells to attach. The medium was replaced on the following day to eliminate non-adherent cells, and the flasks were transferred to a low oxygen atmosphere, 2% O_2_, 5% CO_2_, and 37 °C incubator as described previously by our laboratory [6,21,23]. Media exchange was performed every 3–4 days. Upon 80% confluence, BMSCs were passaged into new T175 flasks (2 × 10^5^ cells/T175) and used up to passage 3 in subsequent experiments. Three BMSC donor populations were used in this study: Donor 1 was a 44-year-old male, Donor 2 was a 24-year-old male, and Donor 3 was a 43-year-old male.

For flow cytometry characterisation of BMSC, cells were trypsinized, washed, and resuspended in flow cytometry buffer containing 0.5% BSA and 2 mM of EDTA in PBS. Cells were distributed into flow cytometry tubes on ice, and fluorescence-conjugated antibodies or isotype controls were added to each tube as per the manufacturer’s instructions. The following antibodies were used: CD45-FITC, CD34-FITC, CD31-PE, HLA-DR-PE, CD105-PE_Cy7, CD44-FITC, CD146-PE, CD271-PE_Cy7 (BD Biosciences, Franklin Lakes, NJ, USA), CD73-BV605, and CD90-BV785 (BioLegend, San Diego, CA, USA). After incubating for 10 min on ice, stained cells were washed and resuspended in flow cytometry buffer and analysed on a FACSymphony A3 flow cytometer (BD Biosciences). Data were analysed using FlowJo v10.9 software (BD Biosciences).

Donors 1, 2, and 3 were characterised for osteogenic and adipogenic differentiation capacity as follows. First, BMSCs were seeded at 6 × 10^4^ cells/cm^2^ in 24-well plates (Nunc, Roskilde, Denmark). The osteogenic induction medium was formulated from high glucose Dulbecco’s Modified Eagle Medium (HG-DMEM) supplemented with 10% FBS, 1% PenStrep, 100 nM of Dexamethasone (Sigma-Aldrich), 50 µM of L-ascorbic acid-2-phosphate, and 10 mM of β-glycerol phosphate (BGP, Sigma-Aldrich). The adipogenic induction medium was formulated from HG-DMEM, 10% FBS, 1% PenStrep, 100 nM of Dexamethasone, 500 µM of 3-isobutyl-1-methylxanthine (IBMX; Sigma-Aldrich), 1 µg/mL of Insulin (Gibco), and 200 µM of Indomethacin (Sigma-Aldrich). The media was exchanged every 3–4 days for 21 days, and then monolayers were fixed with 4% paraformaldehyde (PFA, Sigma-Aldrich) for 30 min at room temperature. Osteogenically induced monolayers were stained with Alizarin Red S (Sigma-Aldrich), and adipogenically induced monolayers were stained with Oil Red O (Sigma-Aldrich), and images were captured with an Olympus BX61 microscope.

**Figure 1:**
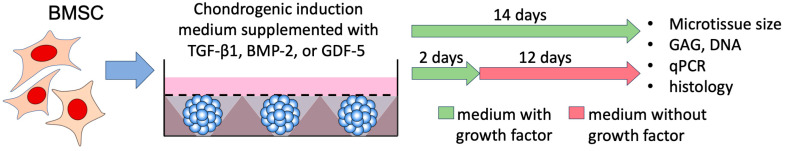
BMSC chondrogenic microtissues cultured in the Microwell-mesh and assays used to characterise the tissue output. BMSCs were assembled into microtissues using the Microwell-mesh culture platform. Microtissues were stimulated with TGF-β1, BMP-2, GDF-5, or no growth factor for 14 days to generate baseline data. Parallel organoid cultures were simulated with TGF-β1, BMP-2, GDF-5, or no growth factor for 2 days followed by culture for 12 days without growth factor. This second group of tissues enabled the assessment of the influence of brief growth factor stimulation on BMSC. The microtissue size was recorded over the culture period, and at the termination of the culture period (14 days) metabolic activity, GAG, DNA, gene expression (using qPCR), and histology were used to evaluate the relative chondrogenic induction achieved with brief or extended stimulation with TGF-β1, BMP-2, GDF-5, or no GF. Image adapted from [21].

For chondrogenic differentiation assays, cells were harvested from expansion cultures and seeded into 12-well Microwell-mesh [23] plates at a seeding density of 0.5 × 10^6^ BMSCs/well (~5000 BMSCs/microwell) in chondrogenic induction medium. BMSCs were forced into the microwells, and cell aggregation was promoted through centrifugation (400× *g* for 3 min), as shown schematically in Figure 1. The chondrogenic induction medium was formulated from high glucose (HG)-DMEM supplemented with 1% PenStrep, 1% insulin–transferrin–selenium–ethanolamine solution (ITS-X), 100 nM of Dexamethasone (Sigma-Aldrich), 200 µM of L-ascorbic acid-2-phosphate (Sigma-Aldrich), 40 µg/mL of L-proline (Sigma-Aldrich), and 1% Sodium Pyruvate. BMP-2 (100 ng/mL, Medtronic, Minneapolis, MN), GDF-5 (100 ng/mL, Peprotech), and TGF-β1 (10 ng/mL, PeproTech) were supplemented during (i) the first two days (brief) followed by chondrogenic induction medium without the growth factor for the remaining 12 days of culture, and (ii) during the entire 14-day (continuous) culture period. In our previous study [6], which tracked BMSC chondrogenesis in response to different numbers of days of TGF-β1 stimulation, we observed that virtually all of the relevant biology was visible in the first 14 days of culture if a microtissue model was used; the differentiation process appeared to be more protracted if a traditional, large-diameter pellet model was used. In the study presented herein, we used only a microtissue model and continued cultures for 14 days, which, based on our previous study, was deemed to be sufficient to interrogate the biology of brief versus continuous growth factor stimulation. In the absence of growth factor, 0.1% BSA in PBS was supplemented. Chondrogenic cultures were incubated at 2% O_2_, 5% CO_2_, and 37 °C. The medium was exchanged every 2 days. Microscopic images were captured, and the microtissue diameter was measured using Fiji software (ImageJ Version 1.53q) [32] to monitor the growth of chondrogenic microtissues.

### 2.2. Characterisation of BMSC Chondrogenic Microtissues

The quantification of DNA and GAG was performed as previously described [23]. Microtissue digestion was initially performed overnight at 60 °C with 125 µg/mL of papain (Sigma-Aldrich) in 100 mM of PBE buffer at pH 6.5. The DNA was quantified using a Quant-iT PicoGreen dsDNA assay Kit (Thermo Fisher Scientific) following the manufacturer’s recommendation. The fluorescence was read in a microplate reader (FLUOstar Omega, Ortenberg, Germany) at 544 nm excitation and 590 nm emission. The GAG was quantified in the papain-digested samples using dimethylmethylene blue (DMMB; Sigma-Aldrich) assay and read at 540 nm with a microplate reader (Multiskan Go; Thermo Fisher Scientific). To estimate the GAG, serial dilutions of chondroitin sulfate derived from shark cartilage were read in parallel to construct the standard curve (Sigma-Aldrich).

The staining procedures started with washing of the microtissues with phosphate-buffered saline (PBS, Gibco) and overnight fixation in 4% PFA. Fixed microtissues were washed again with PBS and embedded in Tissue-Tek optimal cutting temperature compound (OCT, Sakura Finetek, Torrance, CA). Embedded samples were stored at −20 °C prior to use. Cryosectioning of 7 µm sections was performed using Leica Cryostat CM 1950, and sections were collected on poly-lysine-coated slides (Thermo Fisher Scientific). To detect GAG, microtissue sections were gently washed with PBS to remove the OCT, the slides were fixed in 4% PFA for 30 min, and then they were stained with Alcian Blue stain (Sigma-Aldrich) [33] and counterstained with Nuclear Fast Red (Sigma-Aldrich). Specific collagens were detected through immunohistochemistry using the DAB method, as previously reported [21]. In brief, PFA-fixed microtissue sections were treated with 2 mg/mL of hyaluronidase (Sigma-Aldrich), and the tissues were permeabilised with 0.1% Triton X-100 (Sigma-Aldrich) and blocked with 10% normal goat serum (Thermo Fisher Scientific). Sections were stained with the following primary antibodies: anti-collagen type II (ab34712, 1:400) and anti-collagen type X (ab58632, 1:400) from Abcam (Cambridge, England). The primary antibodies were diluted in 1% bovine serum albumin (BSA, Sigma-Aldrich) and incubated at 4 °C overnight. The secondary antibody (1:1000 goat anti-rabbit IgG-HRP, Abcam) was applied the following day and incubated for 1 h at room temperature. Tissue staining was performed using the DAB chromogen kit (Abcam) following the manufacturer’s instructions. Tissue sections were counterstained with Nuclear Fast Red for 5 min and washed with distilled water. Slides were coverslipped using Eukitt mounting medium (Sigma-Aldrich) and imaged with an Olympus IX73 microscope. Control microtissue sections without primary antibodies were used for comparison to confirm specificity of staining.

### 2.3. Quantification of Gene Expression Using qRT-PCR

The total RNA was isolated using the ISOLATE II RNA Mini Kit (Bioline, London, England). Cell lysis was performed with accompanying RLY lysis buffer and treated on-column with DNase-I included in the kit. The RNA concentration and purity were measured using a NanoDrop 1000 spectrophotometer (ThermoFisher). Reverse transcription of mRNA was performed using the SensiFAST cDNA Synthesis Kit (Bioline) to produce cDNA and combined with forward and reverse primers (200 nM) and SYBR Green PCR Master Mix (Applied Biosystems, Waltham, MA). The qRT-PCR run was performed and analysed on a Viia7 Real Time PCR System (Applied Biosystems). The primer sequences are specified in Supplementary Table S1, and GAPDH was used as the housekeeping gene. The run parameters were as follows: a single, initial cycle of 50 °C for 2 min and 95 °C for 10 min, followed by 40 cycles of 95 °C for 15 s and 60 °C for 1 min. The target gene expression relative to the housekeeping gene expression was calculated using the formula:

2^−(Ct(Gene of interest) − Ct(GAPDH)).

### 2.4. Statistical Analysis

All reported quantitative values were expressed as the means of four biological replicate cultures per BMSC donor for each growth factor condition. The values were analysed for normal distribution using the Shapiro–Wilk test (α = 0.05), and it was found that some data sets were not normally distributed. Because data sets could not be presumed to be normally distributed, we used the conservative Kruskal–Wallis and Dunn’s multiple comparison for all analysis. For the comparison of two means, the Mann–Whitney test was used. All statistical analyses were performed in Graph Pad Version 9.2. A *p*-value of less than 0.05 was considered significant.

## 3. Results

### 3.1. Flow Cytometry and Trilineage Differentiation

All three cell donors showed characteristic expression of BMSC-associated surface markers through flow cytometric analysis (Figures S1–S3). BMSCs were negative for haematopoietic cell surface markers CD45, CD34, and HLA-DR and positive for BMSC-associated surface markers CD73, CD90, CD105, CD44 [34], and CD146 [35], and they showed dim expression for CD271 [36]. Donor 2 showed ~21% HLA-DR, but other markers were as typically expected, and so we included these data in this study. Previous studies have noted a similar expression of HLA-DR in clinical-grade BMSC, and the authors suggest that HLA-DR expression should be considered informative rather than as a criterion to define MSC [37]. All three donors underwent osteogenic and adipogenic differentiation, as inferred according to positive Alizarin Red S and Oil Red O staining, respectively (Figure S4). The chondrogenic differentiation capacity of the BMSC is demonstrated extensively in the following sections.

### 3.2. Chondrogenic Microtissues Transiently Exposed to TGF-β1 Displayed Similar Characteristics When Microtissues Were Continuously Treated

Previously, we showed that brief exposure to TGF-β1 during BMSC chondrogenic culture initiation produced microtissues with growth characteristics comparable to those of microtissues cultures that had been continuously exposed to TGF-β1 throughout cultures [6].

**Figure 2:**
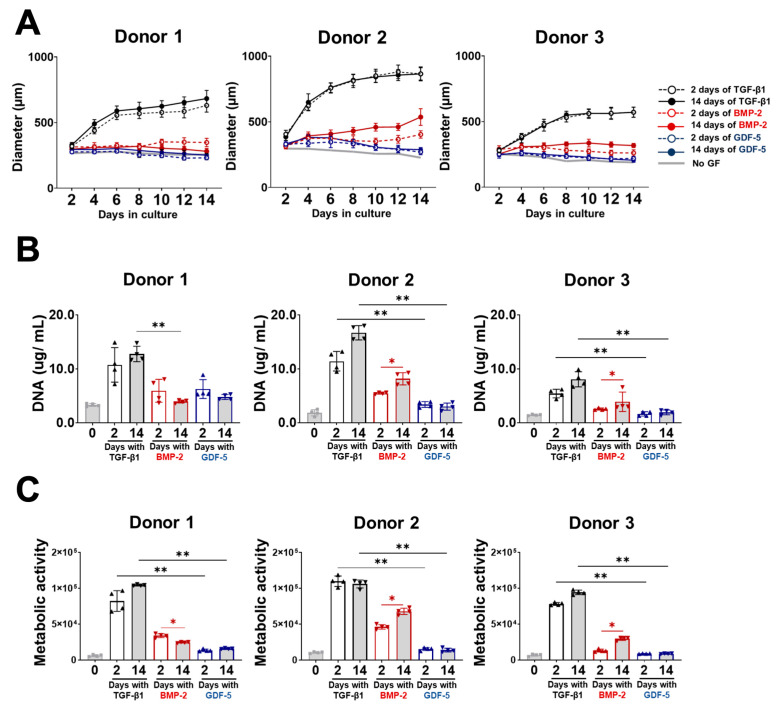
BMSC chondrogenic microtissue growth after brief (2 days) or extended (14 days) exposure to TGF-β1, BMP-2, GDF-5, or no GF. (**A**) Microtissue growth based on diameters. TGF-β1-treated microtissues grew to similar diameters after 2 weeks, regardless of treatment duration (2 or 14 days). This was also true for two of three BMSC donors treated with BMP-2 and all three donors treated with GDF-5. BMP-2- and GDF-5-treated microtissues were significantly smaller in diameter than TGF-β1-treated microtissues. Representative microscopic images of BMSC microtissues are shown in Figure S5. (**B**) DNA content of microtissues. The DNA content of microtissues treated for 2 days or 14 days with TGF-β1 did not differ following the 2-week culture period. Continuous 14 days of BMP-2 treatment resulted in increased DNA content for two out of three donors compared with the brief 2-day treatment. Microtissues treated with BMP-2 and GDF-5 had significantly lower DNA content compared with TGF-β1-treated microtissues for both treatment durations. (**C**) Cellular metabolic activity based on the alamarBlue assay. Similar cell metabolic activity was detected in cultures treated with TGF-β1 for 2 days or 14 days following the 2-week culture period. Metabolic activity was slightly decreased in one out of three donors with extended BMP-2 treatment but increased for the other 2 donors. No difference in metabolic activity was observed with extended GDF-5 treatment. Both BMP-2- and GDF-5-treated microtissues exhibited less metabolic activity compared with TGF-β1, regardless of treatment duration. Statistics, *n* = 4, * *p* < 0.05, ** *p* < 0.01.

Here, we assessed microtissue growth following 2 weeks of culture with brief (2 day) or extended (14 day) supplementation with BMP-2 or GDF-5 and compared these with TGF-β1 and no GF controls (Figure 2 and Figure S5). Microtissue diameter was measured every two days during the two-week culture period (Figure 2A). When cultures were supplemented with TGF-β1, microtissues grew steadily over the 2-week period, with similar diameters regardless of TGF-β1 stimulation for only 2 days or continuously for 14 days. Extended (14 day) BMP-2 treatment improved the growth of microtissues by 1.3-fold and 1.2-fold for Donors 2 and 3, respectively, compared with brief (2 day) treatment; however, these microtissues were substantially smaller (38–44%) than those treated with TGF-β1 (Figure 2A). Neither GDF-5 supplementation duration (2-day nor 14-day) yielded significant microtissue growth, with diameters being similar to those of untreated microtissue controls (no GF) (Figure 2A and Figure S6).

DNA content (Figure 2B) and metabolic activity (Figure 2C) of microtissue cultures were also compared after 2 weeks for the different GF treatments. For TGF-β1-treated microtissues, the DNA content and metabolic activity were unchanged when cultures were supplemented with TGF-β1 for 2 or 14 days (Figure 2B,C). Extended (14 days) BMP-2 supplementation resulted in increased DNA content and metabolic activity in two of three and three of three donors, respectively, when compared with brief (2-day) exposure; however, these were significantly lower than those observed in the TGF-β1 treatments (Figure 2B,C). Brief or extended GDF-5 supplementation of organoid cultures resulted in similar DNA content and metabolic activity at the end of the culture period, and both parameters were significantly lower than those of TGF-β1-treated microtissues (Figure 2B,C).

### 3.3. Brief Exposure to BMP-2 and GDF-5 Facilitates Chondrogenesis but Produces Less GAG Than TGF-β1

**Figure 3:**
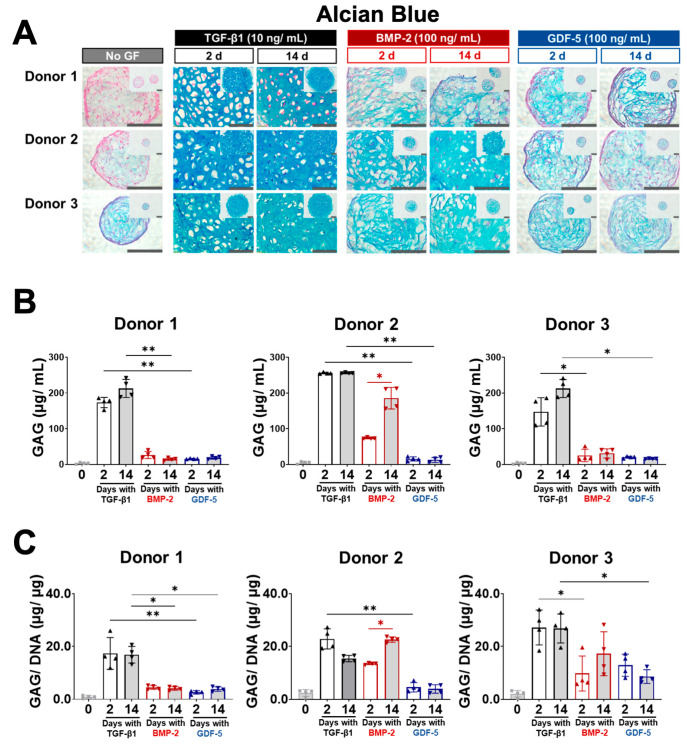
Glycosaminoglycan production in BMSC chondrogenic microtissues after brief (2 days) or extended (14 days) exposure to TGF-β1, BMP-2, GDF-5, or no GF. (**A**) Alcian blue staining after 14-day culture. All GF treatment conditions appeared to variably stimulate GAG accumulation in the microtissue ECM. TGF-β1-treated microtissues had strikingly more GAG staining compared to BMP-2- and GDF-5-treated microtissues. Extended (14-day) BMP-2 treatment appeared to improve GAG staining for Donor 2 compared with the 2-day treatment. GAG production and the appearance of lacunae were generally inferior for BMP-2- and GDF-5-treated microtissues compared with TGF-β1-treated microtissues. Scale bar = 100. (**B**) Quantification of GAG using the DMMB assay. The GAG content of TGF-β1-treated microtissues was unchanged with treatment durations. Extended treatment durations increased the GAG content for one out of three donors in the BMP-2-treated cultures and for two out of three donors in the GDF-5-treated cultures. For both treatment durations, microtissues treated with BMP-2 and GDF-5 produced significantly lower GAG than microtissues treated with TGF-β1. (**C**) GAG relative to DNA content. When GAG was normalised to DNA content, extended treatment with BMP-2 retained a statistically significant difference for Donor 2, but GDF-5 results were no longer statistically significant. Statistics, *n* = 4, * *p* < 0.05, ** *p* < 0.01.

Here, we assessed whether brief (2-day) supplementation of BMSC microtissue cultures with BMP-2 or GDF-5 could induce the production of GAG similarly to brief TGF-β1 treatment [6], or whether extended (14-day) supplementation was beneficial. When treated with TGF-β1 for 2 or 14 days, both yielded similar Alcian blue staining, which is indicative of GAG matrix content, at the end of the 2-week culture (Figure 3A). BMP-2- and GDF-5-cultured microtissues also stained GAG, with Donor 2 appearing to benefit markedly from extended (14-day) BMP-2 treatment compared with the 2-day treatment (Figure 3A). Generally, GAG staining and the appearance of cartilage-like lacunae structures in BMP-2- and GDF-5-cultured microtissues were less prominent relative to TGF-β1 treated microtissues (Figure 3A). Microtissue controls (no GF), which were not supplemented with any GF, appeared to have some faint GAG staining, but this was variable among the three donors (Figure 3A).

GAG content was also estimated in digested microtissues using the DMMB assay (Figure 3B), and these data mirrored the GAG staining seen in histological sections (Figure 3A). Again, TGF-β1 produced the greatest GAG content, regardless of 2 or 14 days of supplementation (Figure 3B). Consistent with Alcian blue staining, only Donor 2 had improved GAG content with extended (14-day) BMP-2 compared with 2-day supplementation (Figure 3B,C). While GAG content appeared to improve slightly for Donors 1 and 2 with extended (14 days) GDF-5 supplementation compared with 2-day supplementation (Figure 3B), statistical significance was lost when GAG content was normalised to DNA content (Figure 3C).

### 3.4. Collagen Type II Production and Chondrogenic Gene Marker Expression after 2 Days and 14 Days of Growth Factor Treatment

**Figure 4:**
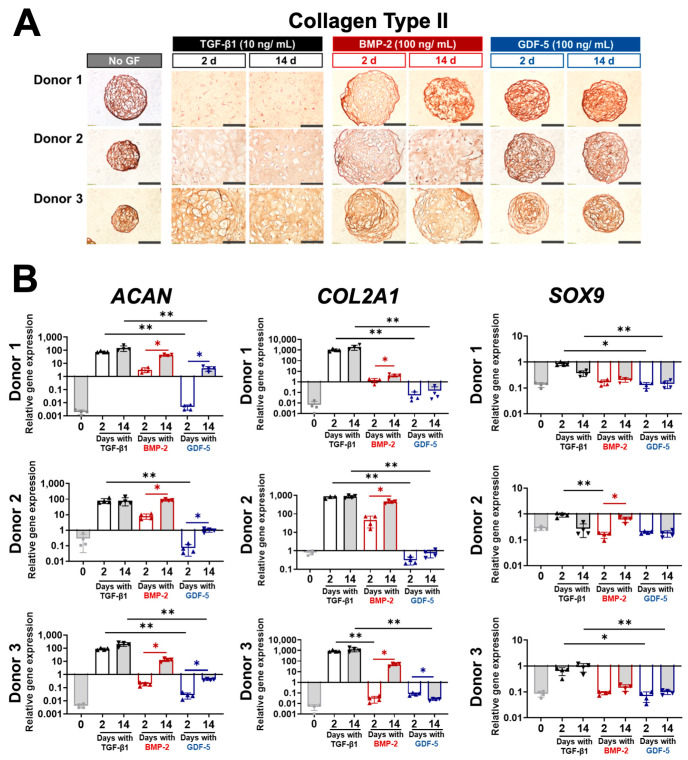
BMSC chondrogenesis after 2 days and 14 days of TGF-β1, BMP-2, GDF-5, and no GF treatment. (**A**) Immunohistochemical staining of collagen type II. While all tissues stained for collagen II, a more diffuse stain correlated with tissues with morphology more similar to native cartilage (GAG-rich matrix and appearance of lacunae; see Figure 3A for GAG stain). Scale bar = 100 µm. (**B**) Gene expression of chondrogenic markers in BMSC microtissues. The potency of the three factors in inducing chondrogenesis was TGF-β1 (for 2 or 14 days) > BMP (for 14 days) > GDF-5 (for 14 days). Statistics, *n* = 4, * *p* < 0.05, ** *p* < 0.01.

We assessed the capacity of brief (2-day) and extended (14-day) BMP-2 and GDF-5 supplementation to induce the expression of chondrogenesis-associated markers and compared these with TGF-β1 and no GF controls. Figure 4A shows all microtissues stained positively for collagen II at the end of the 2-week culture period. BMP-2 and GDF-5 microtissue sections had homogenous but weaker GAG staining (Figure 3A), yet more intense collagen II staining when compared with TGF-β1-treated microtissues. Based on comparison with negative controls, non-specific collagen II staining was ruled out as the cause of more intense collagen II staining (negative controls were tissue sections not exposed to primary antibody, and did not develop any colour after DAB reagent application (Figure S7)). We presume that in microtissue sections where there is less GAG matrix content, the resulting collagen network may as a result be denser, giving a miss-leading indication of matrix maturity. Overall, uniform collagen II staining correlated with GAG-rich staining (Figure 3A) and was similar in TGF-β1 microtissues treated for brief (2-day) and extended (14-day) durations and for Donor 2 when treated with BMP-2 for the extended duration (Figure 4A).

We also used qPCR to evaluate the gene expression of key chondrogenic genes (*ACAN*, *COL2A1*, and *SOX9*) at the end of the 14-day culture period. As we previously reported [6], the expression of chondrogenic genes was similar at day 14 when microtissues were stimulated with TGF-β1 for either brief (2 days) or extended (14 days) periods.

Unlike TGF-β1, microtissues that were stimulated with BMP-2 for an extended period (14 days) did show increased expression of *ACAN* and *COL2A1* when compared with brief (2-day) stimulation (Figure 4B). *ACAN* expression, but not *COL2A1* expression, increased with extended (14-day) GDF-5 treatment compared to brief (2-day) treatment. *SOX9* expression increased in Donors 2 and 3 with prolonged BMP-2 treatment relative to the 2-day treatment, but it was unchanged with prolonged GDF-5 treatment for all three BMSC donors (Figure 4B).

When compared to TGF-β1-treated microtissues, *ACAN* and *COL2A1* gene expression was typically lower in microtissues treated with BMP-2, with the exception of Donor 2, but only when exposed to BMP-2 for the extended 14-day duration (Figure 4B). *ACAN* and *COL2A1* gene expression in GDF-5-treated microtissues was 100 to 1000-fold lower than in TGF-β1-treated microtissues, even with extended exposure (Figure 4B). Based on the expression of key chondrogenic markers, the order of potency of the three GFs to induce chondrogenesis was TGF-β1 (2 days was sufficient) > BMP-2 (improved with extended 14-day treatment) > GDF-5 (low potency with extended treatment).

### 3.5. Brief or Continuous Application of BMP-2 and GDF-5 Did Not Reduce Hypertrophy

**Figure 5:**
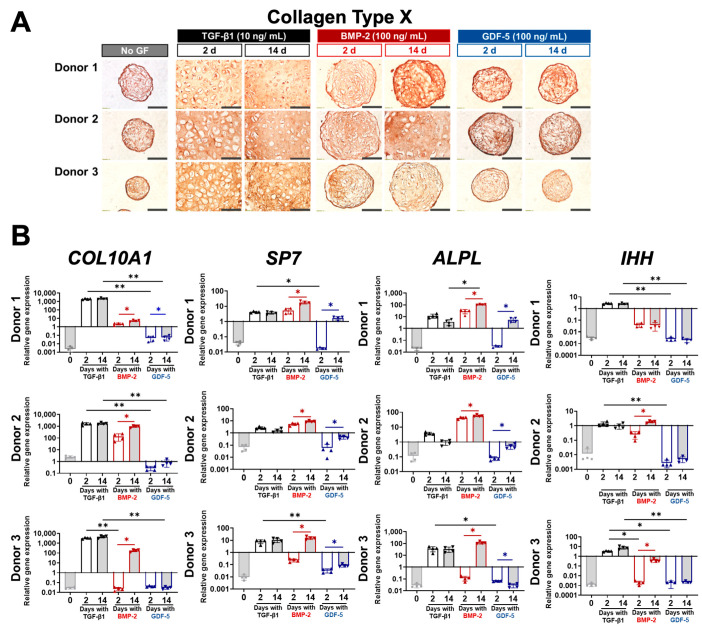
Detection of hypertrophy in BMSC chondrogenic microtissues after 2 days and 14 days of growth factor treatment. (**A**) Immunohistochemical staining of collagen type X was detected in all BMSC chondrogenic microtissue samples, including non-treated controls. No distinct difference was noted in the protein staining of samples treated with 2 days and 14 days of TGF-β1 (three out of three donors), BMP-2 (two out of three donors), or GDF-5 (three out of three donors). Microtissues that were ECM-rich and cell-poor tended to stain more diffusely for collagen X throughout the ECM space, whereas ECM-poor and condensed microtissues exhibited intense collagen X staining around cells. Scale bar = 100 µm. (**B**) Gene expression of hypertrophic markers in BMSC microtissues. Statistics, *n* = 4, * *p* < 0.05, ** *p* < 0.01.

A major limitation of TGF-β1-induced BMSC chondrogenesis is the simultaneous induction of hypertrophy and eventual tissue mineralisation in vivo [6]. Here, we assessed markers of hypertrophy to determine whether BMP-2 and GDF-5 also activated hypertrophic pathways with brief stimulation, similar to TGF-β1. Through immunohistochemistry, the presence of collagen type X was detected in all BMSC microtissues regardless of the growth factor type or treatment duration (Figure 5A). Like the pattern seen in collagen type II staining, smaller and more cellular microtissues tended to have darker collagen X staining due to what we presume is a more condensed network of collagen fibres, as discussed earlier (Figure 4A). Non-specific staining for collagen type X was ruled out by comparison with control microtissue sections not exposed to the primary antibody (Figure S7).

Using qPCR, we also analysed the gene expression of key hypertrophic markers *COL10A1*, *SP7*, *ALPL*, and *IHH* [38,39], which we previously have shown to be upregulated even with a single day of TGF-β1 [6]. Consistent with our previous study, TGF-β1 induced the upregulation of these hypertrophic genes with brief (2-day) and extended (14-day) treatment (Figure 5B). Unlike the temporal effects of TGF-β1 treatment, continuous treatment of microtissues with BMP-2 upregulated the expression of *COL10A1* (three out of three donors), *SP7* (three out of three donors), *ALPL* (three out of three donors), and *IHH* (two out of three donors) compared with microtissues treated with BMP-2 for only 2 days (Figure 5B). Continuous treatment with GDF-5 upregulated *SP7* (three out of three donors) and *ALPL* (two out of three donors), but no significant differences in COL10A1 (two out of three donors) or *IHH* (three out of three donors) gene expression was observed when GDF-5 stimulation was varied from 2 to 14 days (Figure 4B). While more variation was observed between BMSC donors and with treatment duration, BMP-2 did induce the expression of hypertrophic genes. Lower levels of hypertrophic gene expression were observed in GDF-5-cultured microtissues, but this was unremarkable, as GDF-5 was also observed to be a relatively poor inducer of chondrogenesis (Figure 3).

## 4. Discussion

This study sought to understand the temporal potency of skeletally relevant GFs from the TGF-β/BMP superfamily to induce chondrogenesis in BMSC cultures. Conventional BMSC chondrogenic induction cultures are performed by culturing large diameter pellets made from 0.2–1.0 × 10^6^ cells in the presence of TGF-β1 for two or more weeks [40]. Our previous studies uncovered if the pellet diameter is reduced, by culturing BMSC as microtissues, it becomes evident that the TGF-β1-driven differentiation instructions are laid down during the first day of TGF-β1 stimulation [6]. Following the first day of TGF-β1 stimulation, BMSCs appear to proceed simultaneously through chondrogenic and hypertrophic differentiation driven via intrinsic signalling independent of further TGF-β1 stimulation. While it may seem beneficial that BMSC chondrogenesis requires only brief TGF-β1 stimulation, hypertrophy also requires only brief TGF-β1 stimulation, and reducing the TGF-β1 exposure time does not appear to reduce hypertrophic features. If this is a common feature of BMSC behaviour in response to GF stimulation, then modifying the duration of GF treatment, including sequential stimulation with different GFs, may not provide the presumed capacity to modify differentiation outcomes. Several studies have claimed that brief GF treatment led to upregulation of chondrogenic gene markers, enhanced production of the extracellular matrix, and an improved overall mechanical strength of regenerated chondrogenic tissues [41,42,43,44]. The favourable results presented in these studies, however, cannot be fully attributed to the absolute withdrawal of any GF during the 2-week culture [41] or subduing of hypertrophy, as GFs were only removed 2 weeks post differentiation and cultures were continued up to >40 days [42,43,44]. Moreover, the induction medium contained TGF-β1/TGF-β3 [26,41], which we now know will engage intrinsic differentiation machinery following a single day of TGF-β1 stimulation [6]. Thus, previous studies do not provide insight into the effect of brief BMP-2 or GDF-5 stimulation followed by the withdrawal of these GFs. In an effort to better understand how BMSCs respond to stimulation using common chondrogenic induction GFs, we compared BMSCs’ response to brief (2-day) or extended (14-day) treatment with TGF-β1, BMP-2, or GDF-5. We used 10 ng/mL of TGF-β1, as this concentration is sufficiently potent to initiate chondrogenesis in BMSC after as little as one day of treatment in microtissue cultures [6]. For BMP-2 and GDF-5, we used a concentration of 100 ng/mL, as this was the most commonly used or optimal concentration reported for chondrogenic induction in previous studies [17,18,25,26,27,28,29,30,31].

Paralleling our previous study [6], brief (2-day) treatment of BMSC microtissues with TGF-β1 produced the same result as continuous (14-day) TGF-β1 stimulation with respect to microtissue growth and metabolic activity and the induction of chondrogenic markers (GAG production, collagen II protein, and *ACAN*, *COL2A1*, and *SOX9* gene expression) and hypertrophic markers (collagen X protein and *COL10A1*, *SP7*, *ALPL*, and *IHH* gene expression). When the same set of BMSC donors were treated with BMP-2, brief treatment resulted in microtissue growth, but the average diameters of these microtissues were 45–53% smaller than those of similarly TGF-β1-treated microtissues. Extended treatment of cultures with BMP-2 increased the microtissue sizes for two out of three donors, and this was also reflected in the DNA content and metabolic activity. Significant improvement in GAG production and the appearance of lacunae were observed for only one out of three donors with extended BMP-2 treatment, suggesting a variable donor response to BMP-2. Lower GAG production and smaller tissue size have been previously reported with BMP-2 treatment compared with TGF-β1 [45]. Using immunohistochemistry, all microtissues stained positively for collagen II at the end of the 2-week culture period, but the pattern of staining was critical to consider. Notably, regions of microtissues that were rich in GAG exhibited uniform but more diffuse collagen II staining (e.g., TGF-β1-treated tissues). We reason that this staining pattern may be the result of GAG and other ECM molecules “dispersing or diluting” the collagen II network in ECM-rich regions. Conversely, microtissues that had relatively little GAG staining, and were small in diameter, had intense collagen staining in regions that lacked significant GAG staining. The collagen network in poorly induced tissues may be more compacted, thus resulting in more intense collagen staining, but that should not, in this instance, be interpreted as high-quality induction.

Extended BMP-2 stimulation resulted in higher expression levels of chondrogenic genes (*ACAN* and *COL2A1*) and hypertrophic genes (*COL10A1*, *SP7*, and *ALPL*) compared with brief BMP-2 exposure. Compared with TGF-β1-treated cultures, extended stimulation with BMP-2 resulted in higher levels of osteogenic-associated gene expression (*SP7* and *ALPL*). As with TGF-β1, where chondrogenic genes were observed to be upregulated by BMP-2, so were hypertrophic/osteogenic genes, suggesting that these two pathways are likely coupled in BMSC or at least in response to these GFs. When supplied continuously, BMP-2 stimulation alone can drive chondrogenesis, albeit with reduced potency compared to TGF-β1, and potentially at the expense of higher osteogenic induction.

As mentioned, the major limitation of BMSC-derived cartilage tissue engineering is hypertrophic differentiation, which results in a non-ideal tissue composition and a propensity for this tissue to be remodelled and mineralise when implanted in vivo [6]. We included GDF-5 in our study because of its importance during cartilage and synovial joint development [46] and given that a recent study reported enhanced chondrogenesis with the absence of hypertrophy in GDF-5-induced BMSC cultures [19]. Across all three BMSC donors, we found that GDF-5 was the least potent inducer of BMSC chondrogenesis, thus promoting the smallest growth in microtissue diameter, DNA content, metabolic activity, and GAG production and showing the smallest changes in gene expression when compared with TGF-β1 and extended BMP-2 treatment. Morphologically, GDF-5-treated microtissues showed reduced staining for GAG, and the tissues appeared condensed, with less ECM between cells. Extended treatment with GDF-5 did not show improvement in microtissue growth nor GAG production for any of the three BMSC donors tested compared with brief exposure. The lack of significant microtissue growth and GAG content using GDF-5 in our studies is in contrast with a previous study using rat adipose-derived stromal cells [18]. Based on gene expression, extended GDF-5 treatment increased the expression of *ACAN* consistently among BMSC donors, but the levels observed in these tissues were much lower than those of TGF-β1-treated tissues, which is consistent with two previous studies [25,30] and in contrast to another [19]. GDF-5 stimulation did not upregulate *COL2A1* to the levels seen in TGF-β1- or BMP-2 (extended)-treated tissues, which is consistent with the report from Clarke et al. [25]. Murphy et al. reported similar *COL2A1* gene expression in GDF-5 treated pellets with TGF-β1, but they observed lower expression compared to BMP-2 [30]. In our cultures, GDF-5 treatment did not upregulate *COL10A1* gene expression with continuous exposure, but it induced the expression of other osteogenesis-associated genes (*SP7* and *ALPL*). While a significant increase in *COL10A1* expression was not observed with even extended GDF-5 treatment, this may be due to the poor overall response of these BMSC donors to GDF-5, rather than the ability of GDF-5 to induce chondrogenesis without also inducing hypertrophy, as suggested previously [19]. When used on chondrocytes, GDF-5 was reported to induce significant matrix formation, stimulate *ACAN* and *SOX9* gene expression, and downregulate matrix-degrading enzymes [20,47], suggesting that the chondrogenic effect of GDF-5 may be enhanced to a greater extent in cell types other than BMSCs. Lastly, comparison of chondrogenic and hypertrophic gene expression observed in BMSC microtissue relative to previous studies that used pellet culture models may confounded by relative tissue size. Pellet cultures, due to their relatively large diameter, suffer from diffusion gradients which contribute to radially heterogenous tissue formation and perceived slower differentiation kinetics [6]. By contrast, smaller diameter microtissues yield more homogenous tissue and differentiation kinetics may appear to be accelerated. To avoid this confounding variable, studies may require replication in a microtissue model. 

## 5. Conclusions

The GFs we tested in this study (TGF-β1, BMP-2, and GDF-5) induced chondrogenesis, but with differing temporal potency and BMSC donor response. BMSC microtissues reached a similar end point with brief or continuous TGF-β1 stimulation. In BMP-2-treated cultures, BMSC chondrogenic induction was significantly increased with extended BMP-2 treatment. In GDF-5-treated cultures, BMSC chondrogenic induction was also increased with extended stimulation, but the overall response to this GF was the lowest. In summary, these data show that (1) different GFs can induce chondrogenesis with variable temporal potencies (TGF-β1 > BMP-2 > GDF-5), thus requiring different durations of stimulation, (2) chondrogenic pathways appear to be coupled or intertwined with hypertrophic pathways in BMSC in response to these GFs, and (3) with less potent GFs (BMP-2 and GDF-5), BMSC variability appears to be amplified.

While there are some similarities in the mode of action of TGF-β1, BMP-2, and GDF-5, there are also some differences, and it is possible that these differences could be exploited to generate cartilage-like tissue that does not have a propensity to undergo hypertrophy. TGF-β1 signals through Smad 2/3, but it can phosphorylate Smad 1/5/9 when a different ALK receptor is used [48]. BMP-2 and GDF-5 signal through Smad 1/5/9 to regulate chondrogenesis and activate chondrocyte terminal differentiation [26,49,50]. Commonly, strategies propose to counter BMSC hypertrophy include targeting the Smad 1/5/9 pathway [51], although complete blockade of this pathway at the onset of differentiation also impairs cartilage development [52]. In our own studies, we found that the obstruction of BMP signalling dampened hypertrophy, but at the expense of chondrogenic differentiation [21]. Cumulatively, these data highlight that chondrogenic and hypertrophic differentiation are intertwined, and they also emphasise the challenge of modulating one independently of the other. With single cell RNA-Seq becoming increasingly available, and if it becomes less expensive, future studies might aim to map gene expression cascades evolving from different combinations of growth factor stimulations and hypertrophic blockades. Using this approach, it may be possible to identify a stimulation strategy that induces chondrogenesis while effectively dampening hypertrophy.

## Data Availability

The data presented in this study are available on request.

## Supplementary Materials

The following supporting information can be downloaded at: https://www.mdpi.com/article/10.3390/cells13010037/s1, Table S1 includes information related to the primer sequences described in the methods for qRT-PCR reactions; Figures S1–S3 provide flow cytometry characterisation data for BMSC Donors 1-3; Figure S4 provides osteogenic and adipogenic differentiation characterisation data for BMSC Donors 1–3. Figure S5 includes information related to Figure 2. Microscopic images of BMSC microtissues with transient (2 days) and continuous (14 days) supplies of TGF-β1, BMP-2, and GDF-5; Figure S6 includes information related to Figure 2A. Comparison of microtissue diameters after 2-week culture; Figure S7 includes information related to Figure 4 and Figure 5. Microscopic images of sample BMSC microtissue sections stained with DAB method treated with collagen type II and collagen type X primary antibodies compared to sections without primary antibody treatment.
