# Supplementary material for: Microtissue Culture Provides Clarity on the Relative Chondrogenic and Hypertrophic Response of Bone-Marrow-Derived Stromal Cells to TGF-β1, BMP-2, and GDF-5
Source: Cells. 2023 Dec 23;13(1):37. doi: 10.3390/cells13010037 (PMC10778331; doi:10.3390/cells13010037)
Supplement: Supplementary file 1 [file cells-13-00037-s001.zip › cells-2647953-supplementary.pdf]

Gene		Sequence (5' - 3')
**Housekeeping gene**		
GAPDH	F	ATGGGGAAGGTGAAGGTCG
	R	TAAAAGCAGCCCTGGTGACC
**Chondrogenic genes**		
COL2A1	F	GGCAATAGCAGGTTCACGTACA
	R	CGATAACAGTCTTGCCCCACTT
ACAN	F	TCGAGGACAGCGAGGCC
	R	TCGAGGGTGTAGCGTGTAGAGA
SOX9	F	ACTCCTCCTCCGGCATGAG
	R	GCTGCACGTCGGTTTTGG
**Hypertrophic genes**		
COL10A1	F	ACTCCCAGCACGCAGAATCCA
	R	TGGGCCTTTTATGCCTGTGGGC
SP7	F	TTCTGCGGCAAGAGGTTCACTC
	R	GTGTTTGCTCAGGTGGTCGCTT
ALPL	F	CGTGGCTAAGAATGTCATCATGTT
	R	TGGTGGAGCTGACCCTTGA
IHH	F	ATGAAGGCAAGATCGCTCG
	R	GATAGCCAGCGAGTTCAGG

Table S1. Table S1 includes information related to the primer sequences described in the methods for qRT-PCR reactions.

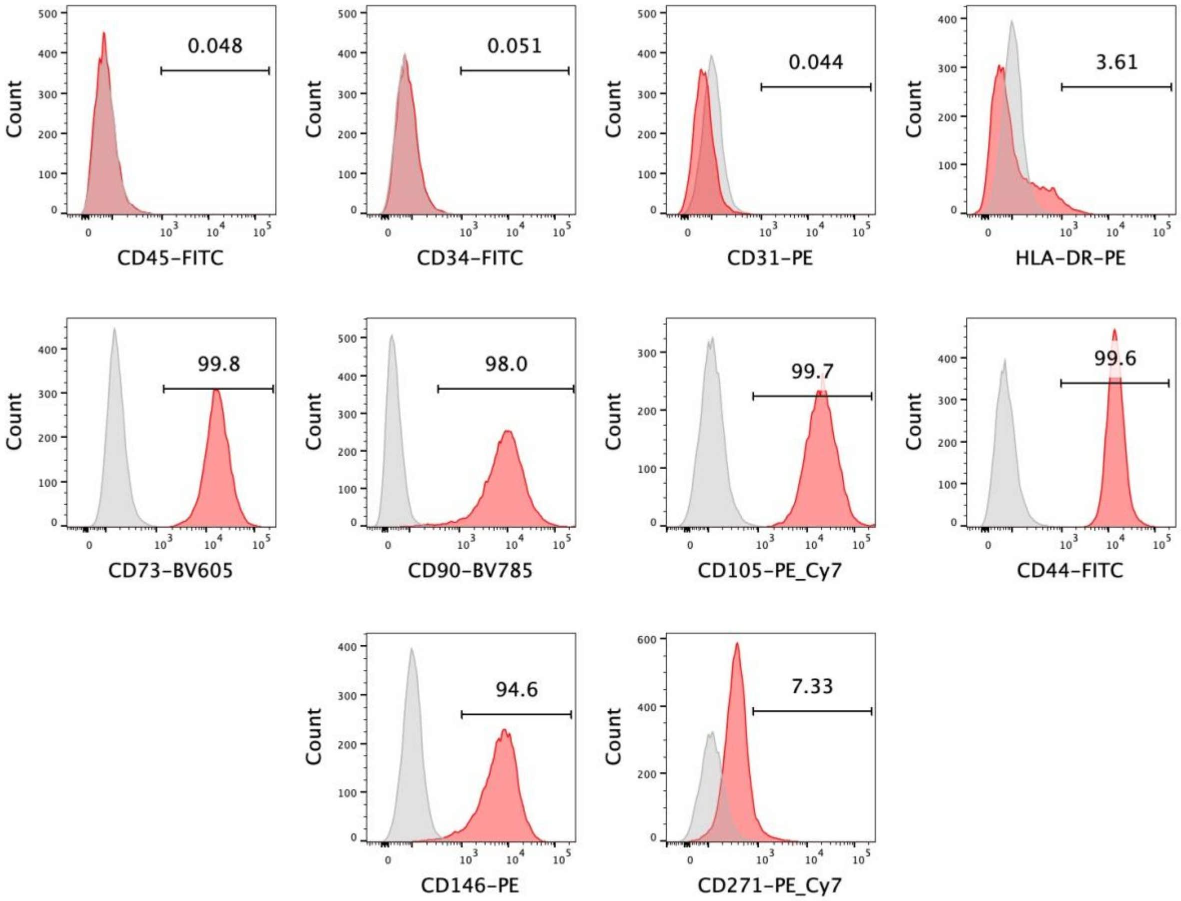

Figure S1. BMSC donor 1 flow cytometry characterisation. Cells were negative for haematopietic cells surface markers, CD45, CD34 and HLA-DR (<5 %) and positive for BMSC-associated surface markers, CD73, CD90, CD105, CD44, and CD146; cells were dim for CD271. Antibody-stained cells are represented by red histograms and isotype controls are grey.

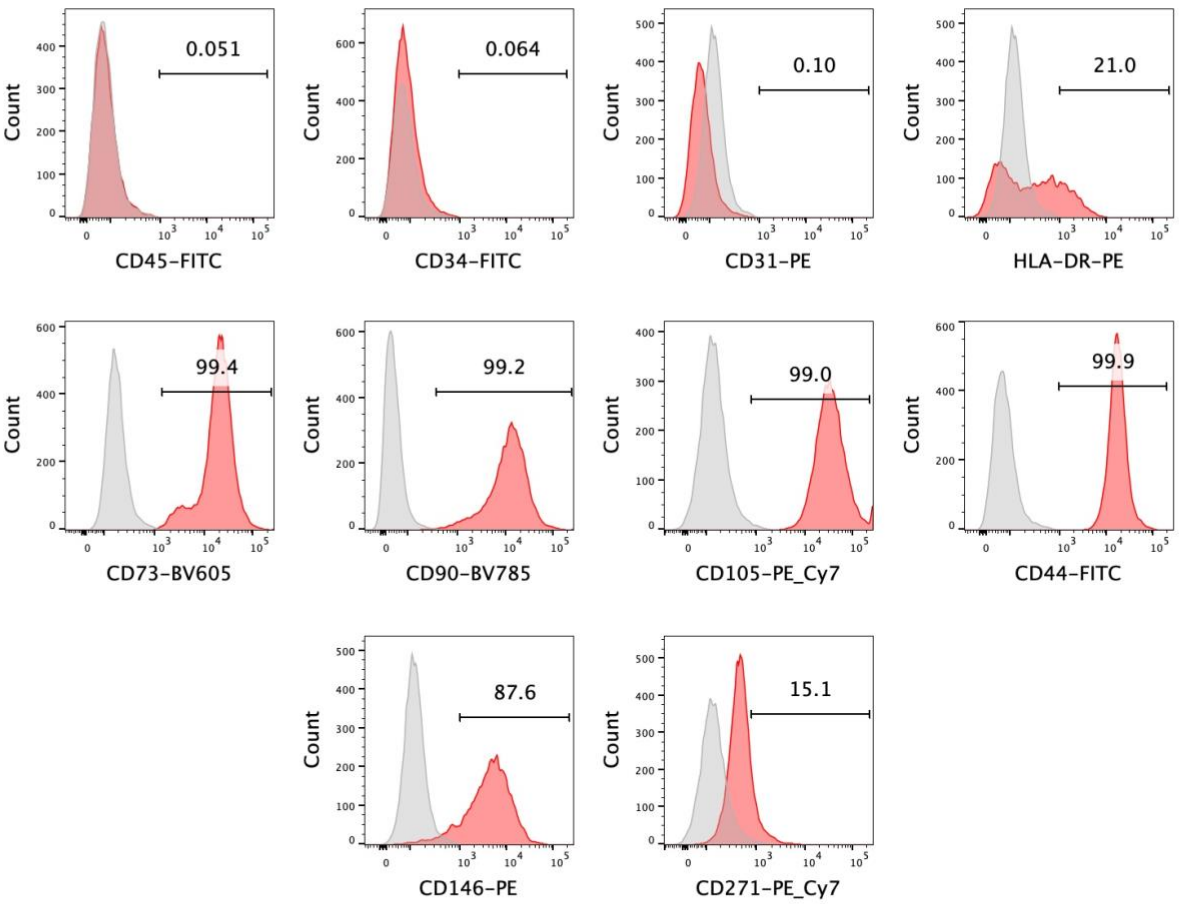

Figure S2. BMSC donor 2 flow cytometry characterisation. Cells were negative for haematopietic cells surface markers, CD45, CD34 and HLA-DR (~21 % positive) and positive for BMSC-associated surface markers, CD73, CD90, CD105, CD44, and CD146; cells were dim for CD271. Antibody-stained cells are represented by red histograms and isotype controls are grey.

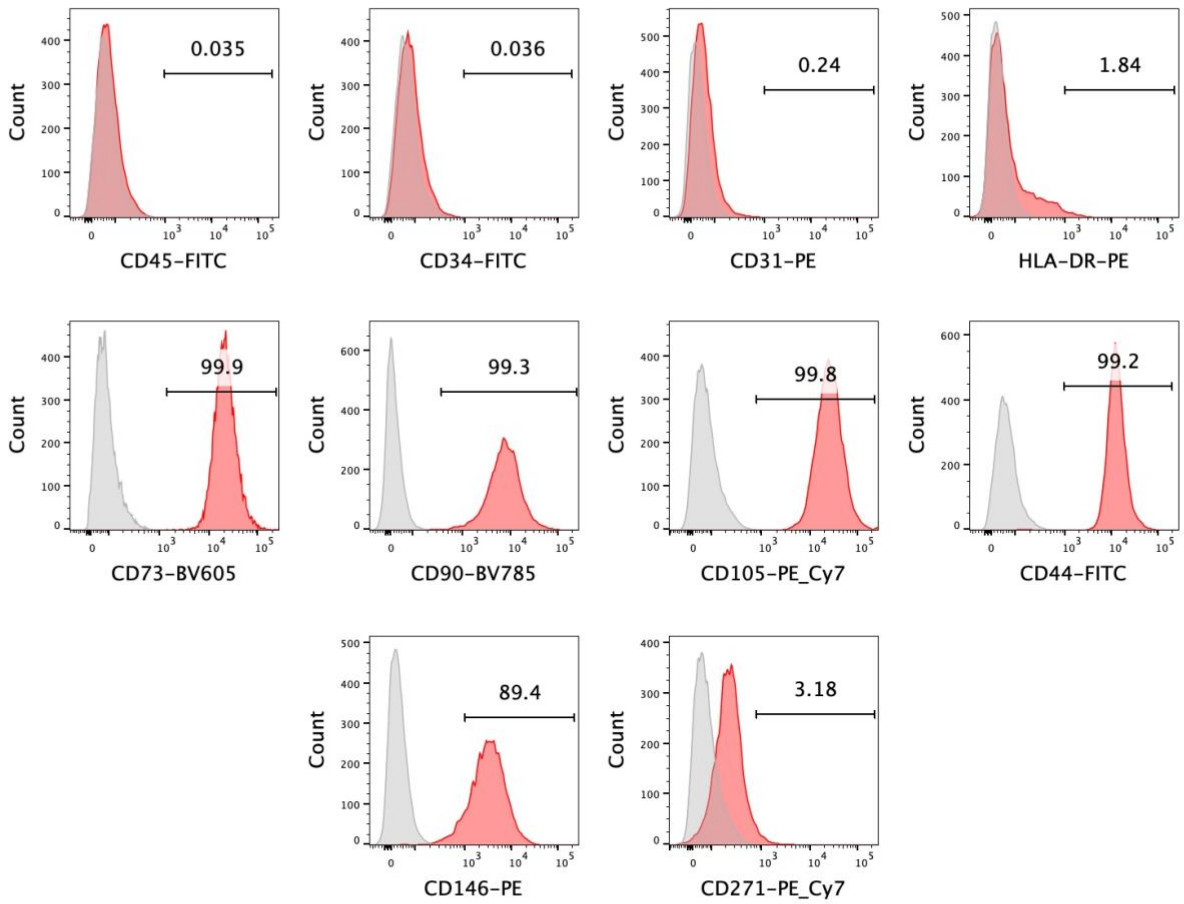

Figure S3. BMSC donor 3 flow cytometry characterisation. Cells were negative for haematopietic cells surface markers, CD45, CD34 and HLA-DR (<5 % positive) and positive for BMSC-associated surface markers, CD73, CD90, CD105, CD44, and CD146; cells were dim for CD271. Antibody-stained cells are represented by red histograms and isotype controls are grey.

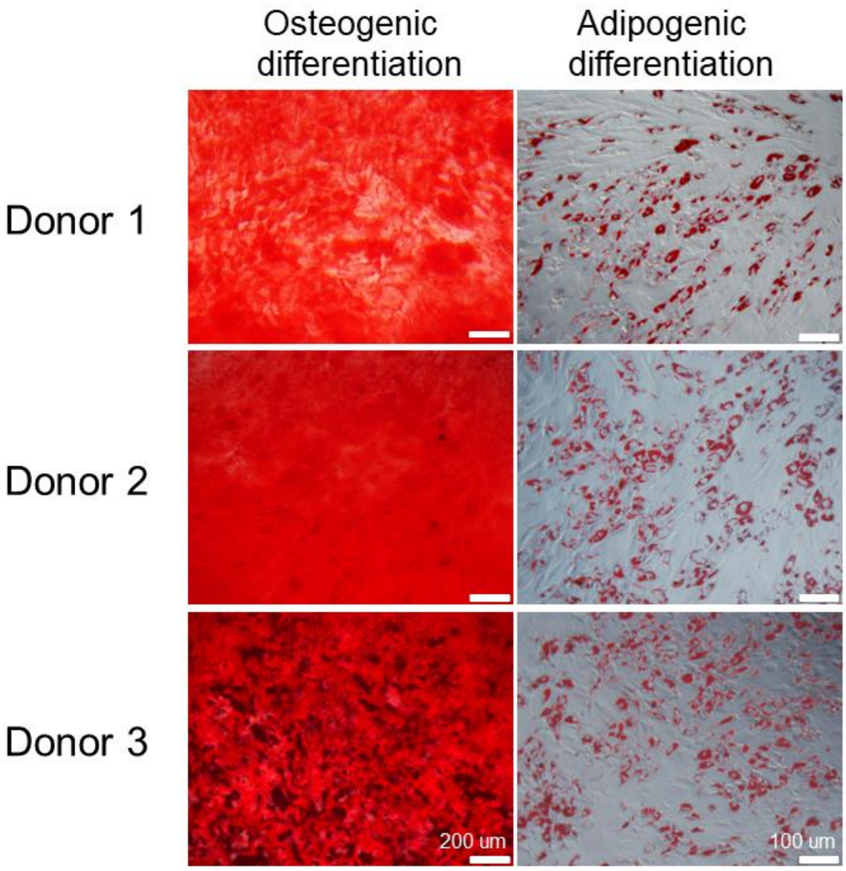

Figure S4. Osteogenic and Adipogenic differentiation. Left column, 21-days osteogenic induction of BMSC monolayers followed by staining with Alizarin Red S (scale bars = 200 μm). Right column, 21-days adipogenic induction of BMSC monolayers followed by staining with Oil Red O (scale bars = 100 μm).

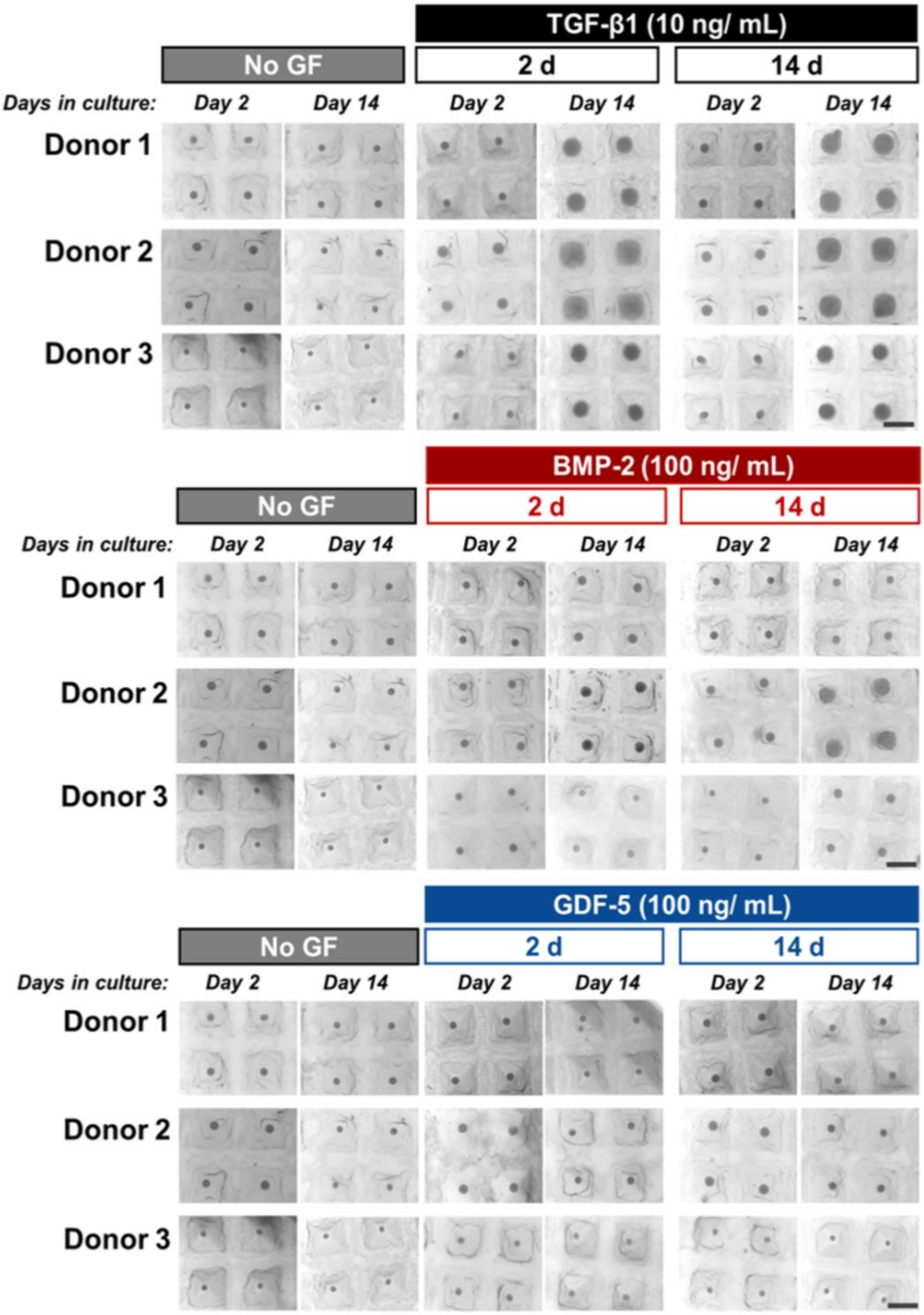

Figure S5. Figure S1 includes information related to Figure 1. Microscopic images of BMSC microtissues with transient (2 days) and continuous (14 days) supply of TGF-β1, BMP-2, and GDF-5.

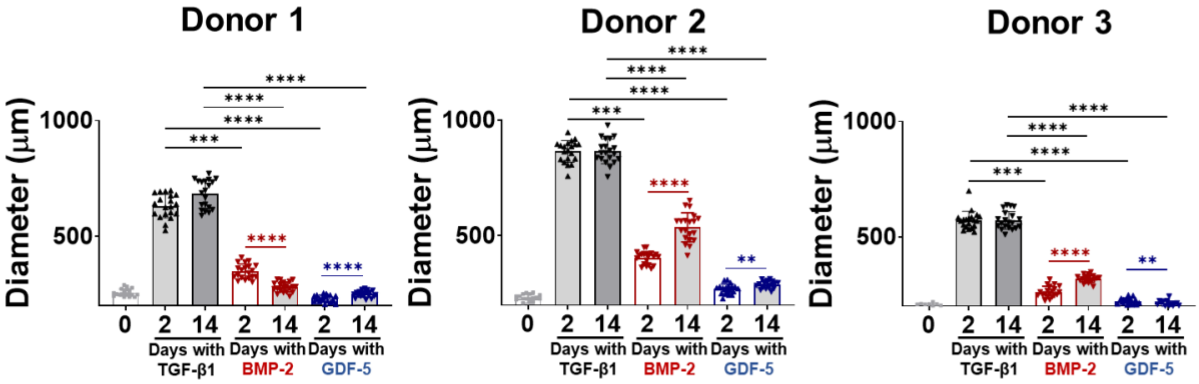

Figure S6. Figure S2 includes information related to Figure 1A. Comparison of microtissue diameters after 2-week culture.

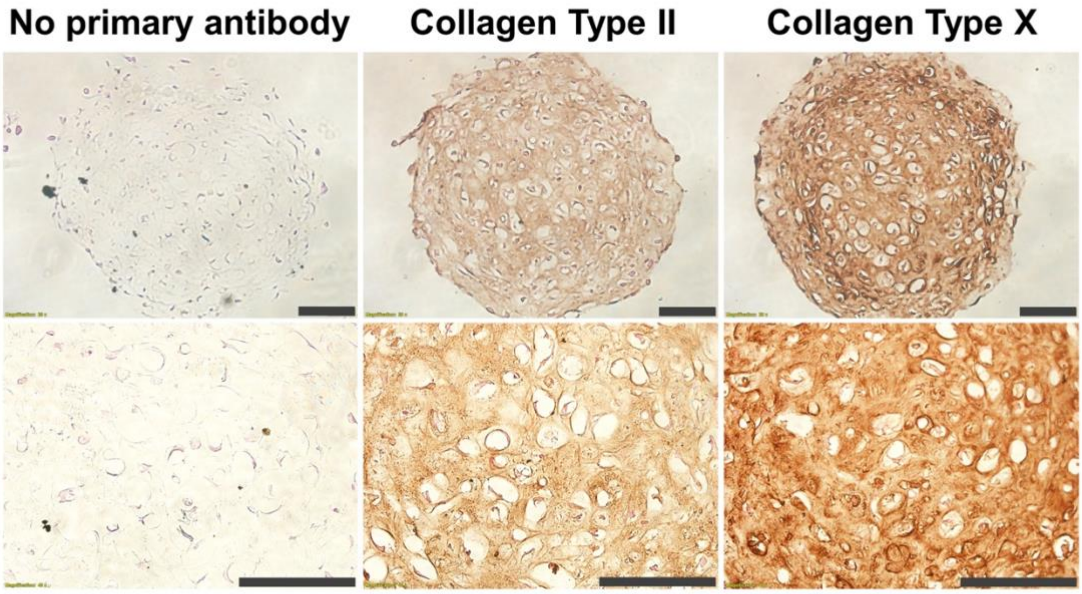

Figure S7. Figure S3 includes information related to Figure 3-4. Microscopic images of sample BMSC microtissue sections stained with DAB method treated with collagen type II and collagen type X primary antibodies compared to sections without primary antibody treatment. Scale bar = 100 µm
